# Wide-Field Landers Temporary Keratoprosthesis in Severe Ocular Trauma: Functional and Anatomical Results after One Year

**DOI:** 10.1155/2015/163675

**Published:** 2015-11-04

**Authors:** Katarzyna Nowomiejska, Dariusz Haszcz, Cesare Forlini, Matteo Forlini, Joanna Moneta-Wielgos, Ryszard Maciejewski, Tomasz Zarnowski, Anselm G. Juenemann, Robert Rejdak

**Affiliations:** ^1^Department of General Ophthalmology, Medical University of Lublin, Lublin, Poland; ^2^Department of Ophthalmology, Santa Maria Delle Croci Hospital, Ravenna, Italy; ^3^Institute of Ophthalmology, University of Modena, Modena, Italy; ^4^Human Anatomy Department, Medical University of Lublin, Lublin, Poland; ^5^Department of Glaucoma Diagnostics and Microsurgery, Medical University of Lublin, Lublin, Poland; ^6^Department of Ophthalmology, University Eye Hospital, Rostock, Germany; ^7^Department of Experimental Pharmacology, Medical Research Centre, Polish Academy of Sciences, Warsaw, Poland

## Abstract

*Purpose*. To evaluate longitudinal functional and anatomical results after combined pars plana vitrectomy (PPV) and penetrating keratoplasty (PKP) using a wide-field Landers intraoperative temporary keratoprosthesis (TKP) in patients with vitreoretinal pathology and corneal opacity due to severe ocular trauma. *Material and Methods*. Medical records of 12 patients who had undergone PPV/PKP/KP due to severe eye trauma were analyzed. Functional (best-corrected visual acuity) and anatomic outcomes (clarity of the corneal graft, retinal attachment, and intraocular pressure) were assessed during the follow-up (mean 16 months). *Results*. Final visual acuities varied from NLP to CF to 2 m. Visual acuity improved in 7 cases, was unchanged in 4 eyes, and worsened in 1 eye. The corneal graft was transparent during the follow-up in 3 cases and graft failure was observed in 9 eyes. Silicone oil was used as a tamponade in all cases and retina was reattached in 92% of cases. *Conclusions*. Combined PPV and PKP with the use of wide-field Landers TKP allowed for surgical intervention in patients with vitreoretinal pathology coexisting with corneal wound. Although retina was attached in most of the cases, corneal graft survived only in one-fourth of patients and final visual acuities were poor.

## 1. Introduction

Severe ocular trauma is usually associated with combined anterior and posterior segment damage and is a surgical challenge. Many eyes (about 50%) with perforating injuries have entrance wound in the cornea [1]. Small and thin corneal scars make it possible to perform pars plana vitrectomy (PPV). However, the view of the retina and vitreous may be difficult due to massive corneal epithelium edema, scar formation, blood staining, or neovascularization of the cornea. Thus, it may be not easy to attach the retina, shave the vitreous body, or remove intraocular foreign body (IOFB) during PPV. Endoscopy-assisted PPV is one option in these cases [2]; however it requires specialized equipment and training (steep learning curve).

Development of temporary keratoprosthesis (TKP) allows performing elaborated surgery adequately in severely traumatized eyes requiring PPV with an opaque cornea.

**Figure 1:**
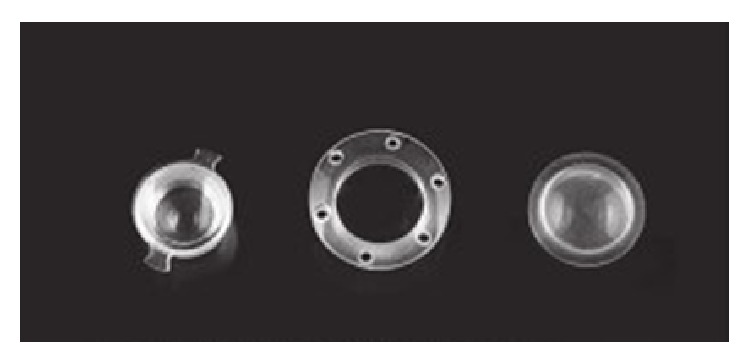
Original Landers PMMA keratoprosthesis (left), wide-field Landers PMMA keratoprosthesis (middle), and Eckart silicone keratoprosthesis (right). Picture from a book by Narendran et al. [5].

Use of TKP during PPV was first described by Landers et al. in 1981 [3]. The first TKP was biconcave optical cylinder 5 mm in length made of polymethyl methacrylate (PMMA) and had 7.2 mm in diameter (Figure 1). In 1987 Eckardt [4] proposed a modification of TKP made of silicone and having a diameter of 10 mm of the outer cylinder and a diameter of 7 mm of the inner cylinder with 2.8 mm in length. However, Eckardt TKP became cloudy after reusing as there were no holes for sutures and the silicone became damaged after multiple suture trucks [5]. Moreover, the view into the peripheral retina was limited. The modification is Landers third-generation wide-field TKP which is hard plastic PMMA device with a 1 mm cylinder protruding to the anterior chamber (choice of diameters: 6.2 mm, 7.2 mm, or 8.2 mm) with a mushroom-shape corneal surface of a diameter 15.5 mm and 6 suture holes in the periphery [5]. Landers wide-field TKP is durable and reusable and can be sutured firmly to the globe; moreover it has a convex anterior surface to facilitate viewing to the posterior pole and periphery of the retina [5]. The latest improvement is trunkless Landers wide-field TKP with no central trunk extending down into the opening in the cornea [6]. Additionally, there are models including 20 G stainless steel infusion line [6]. Thus, the major differences between three generations of TKPs are material (PMMA or silicone) and dimensions of the cylinder and the corneal surface to have a better view to the retina.

The aim of this study was to show functional and anatomical results after one year in patients after severe ocular trauma treated with combined surgery: PPV with wide-field Landers TKP and PKP.

## 2. Methods

**Figure 2:**
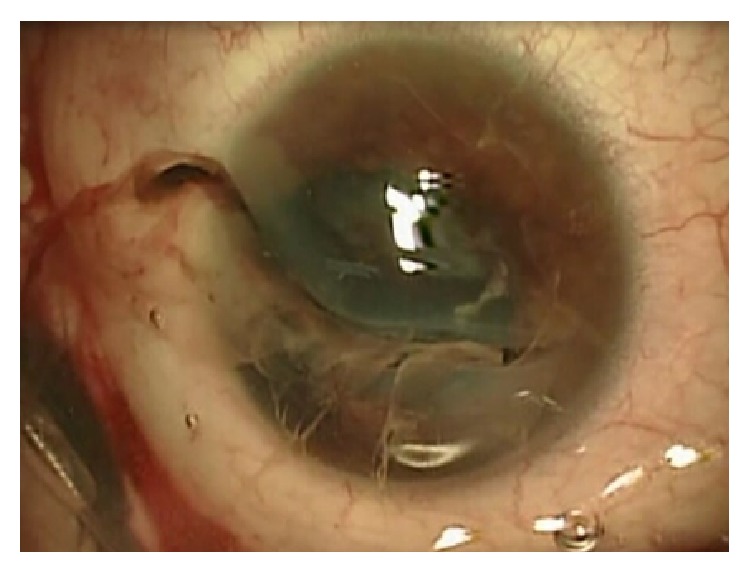
Extended corneal laceration (zone II) with iris and vitreous prolapse due to penetrating trauma: case number 3.

**Figure 3:**
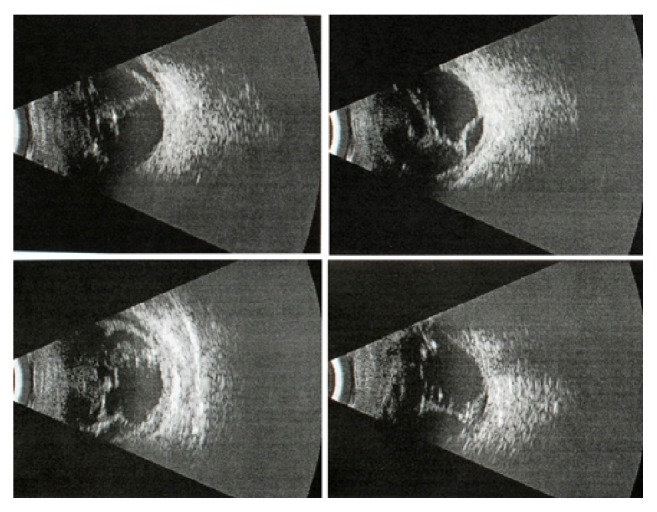
Retinal detachment seen in ultrasonography in patient with corneal laceration.

This interventional case series included patients, who had combined PPV and TKP and PKP surgery performed between March 2009 and December 2011 in the Department of General Ophthalmology of Medical University of Lublin, Lublin, Poland. The study followed the tenets of the Declaration of Helsinki. The patients gave their written informed consent. The inclusion criteria were as follows: (1) severe open eye injury with corneal or corneoscleral laceration, (2) retinal detachment, (3) combined PPV/TKP/PKP surgery, and (4) follow-up of at least 10 months. In all cases corneal wound (Figure 2) was sutured during the first operation (Figure 3) as an emergency procedure; TKP/PKP/TPPV was performed as a second surgery, few days later (mean period of 24 days).

The patient data were recorded including age, gender, visual acuity, intraocular pressure, and history of ocular injuries. Ocular trauma data included the mechanism and type of injury classified according to the Ocular Trauma Classification [7, 8]. All included eyes underwent open-globe injury (injury with full thickness wound to the cornea or/and sclera), penetrating, IOFB, or eye rupture. The site of the injury was classified as zones I, II, and III depending on the location of the wound within the cornea or sclera. Zone I injuries are confined strictly to the cornea, zone II injuries involve the anterior sclera of 5 mm from the limbus, and zone III injuries involved full thickness scleral defects more posterior than 5 mm from the limbus [7, 8].

**Figure 4:**
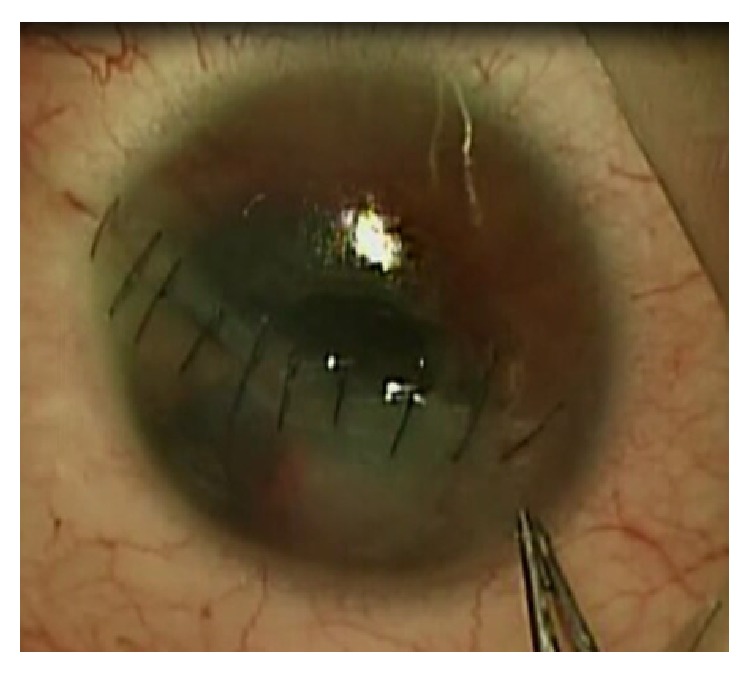
Sutured corneal wound during an emergency operation.

As initial and final visual acuities were poor and not measured using Snellen charts, hand motion (HM), finger counting (FC), light perception (LP), and no light perception (NLP) forms were used to describe visual acuities of included patients. The preoperative examination included visual acuity, intraocular pressure, and B-scan ultrasonography (Figure 4). The follow-up examination included visual acuity, intraocular pressure, corneal graft transparency, and retinal attachment.

The mean follow-up period was 16 months (range 10–29 months). The schedule of control examinations after surgery was planned as follows: next day, one week, one month, three months, six months, one year, one and half of a year and two years after operation.

Functional (best-corrected visual acuity: improved, stable, and worsened) and anatomic outcomes (clarity of the corneal graft, retinal attachment, and intraocular pressure) were assessed during the follow-up.

## 3. Surgical Technique

All surgeries were performed under general anesthesia by two surgeons, anterior segment surgeon and vitreoretinal surgeon. Mean operation time was 3 hours (range 2–4 hours).

**Figure 5:**
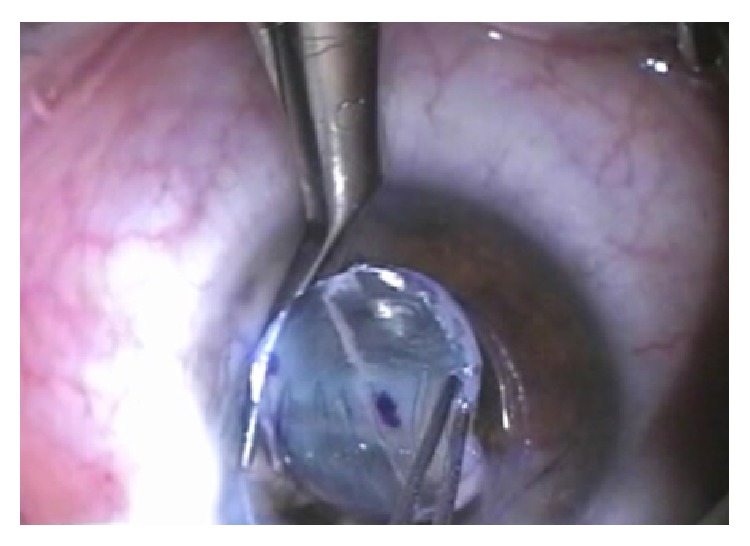
Trephining the cornea during combined vitrectomy/penetrating keratoplasty/temporary keratoprosthesis surgery.

**Figure 6:**
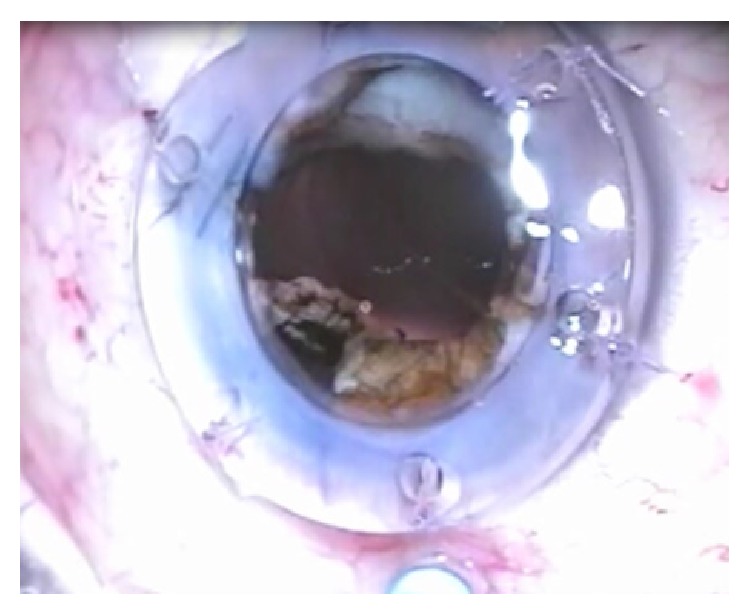
Wide-field temporary keratoprosthesis sutured to the corneal bed using six Vicryl sutures.

**Figure 7:**
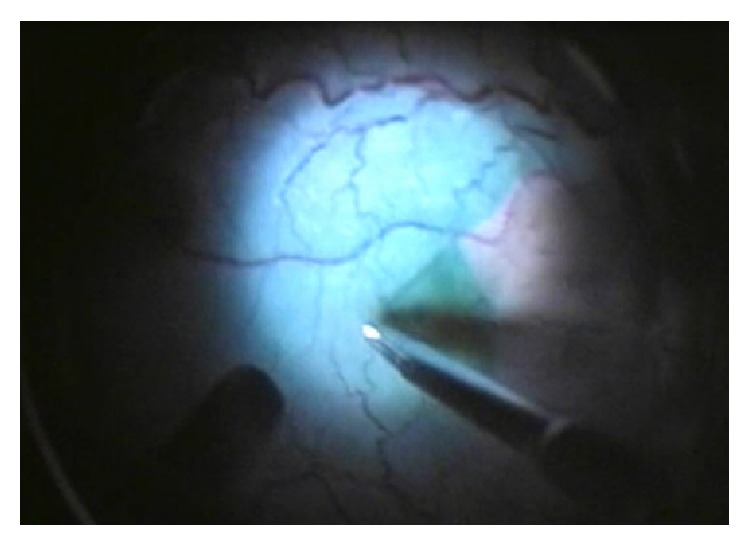
Internal limiting membrane peeling visualized through the temporary keratoprosthesis after dying with indocyanine green during vitrectomy.

Anterior chamber maintainer was first put into anterior chamber. Next, corneal trephination was performed (Figure 5) using handheld trephine and microcorneal scissors (diameter of the corneal button 7.5 mm). The traumatic cataract was then removed with phacoemulsification and next a scleral fixation of artificial intraocular lens was performed. The Landers wide-field TKP (Ocular Instruments, Bellevue, WA) with 1 mm trunk was then placed into the corneal bed and sutured to the limbus using Vicryl 8.0 sutures (Figure 6). Infusion cannula was put to the vitreous cavity and visualized. Additional sclerotomies for the cutter and light pipe were done around the TKP to perform 23-gauge (G) PPV (Constellation, Alcon, Fort Worth, Texas) including posterior vitreous detachment and shaving of the vitreous base. After dying with indocyanine green, internal limiting membrane (ILM) peeling was done (Figure 7). Perfluorocarbon liquids were used to attach the retina. At the end of the operation TKP was removed and the donor cornea button (diameter 7.75 mm) was sutured in place using Nylon 10.0 full thickness sutures after removal of the TKP. Next, fluid-air and air-silicone oil (5 000 cSt) exchange was done. The sclerotomies were closed with Vicryl 7.0 sutures.

## 4. Results

The inclusion criteria were met by 12 patients (Table 1). The mean age of included patients was 42 years (range 21–71 years); there were 10 males and 2 females. As a mechanism of trauma (Birmingham Eye Trauma Terminology (BETT) scale) there were 7 lacerations, IOFB was present in four cases, and there was one eye rupture. Wound location was classified according to Ocular Trauma Classification Group as zone I in 5 eyes, as zone II in 5 eyes, and as zone III in 2 eyes. Visual acuities at presentation were as follows: 4 NLP eyes, 2 light perception eyes, HM in 5 eyes, and CF in 1 eye. There was an improvement of the visual acuity in 7 eyes (59%), no change in 4 (33%) eyes, and worsening in 1 case (8%). There were three NLP eyes that did not improve in the visual acuity. One revealed hypotony, one phthisis bulbi. Final visual acuities were as follows: 4 NLP eyes, 1 HM eye, and 7 CF eyes, the best CF to 2 m (case 3).

**Figure 8:**
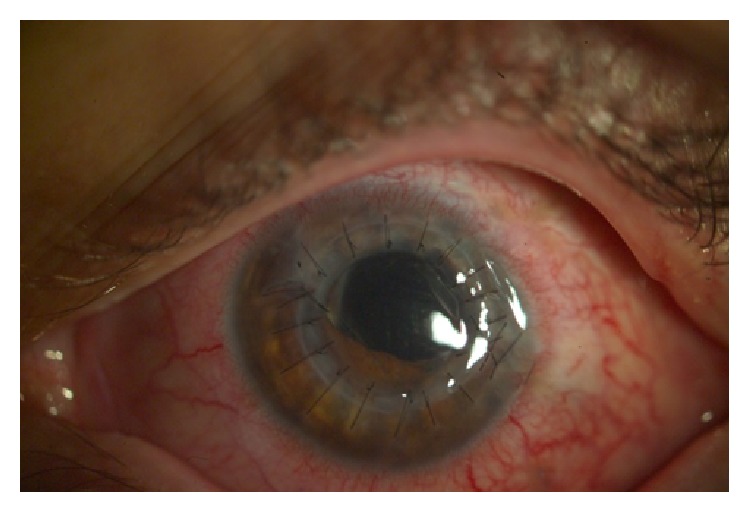
The eye after one month of the follow-up after combined surgery, clear corneal graft.

**Figure 9:**
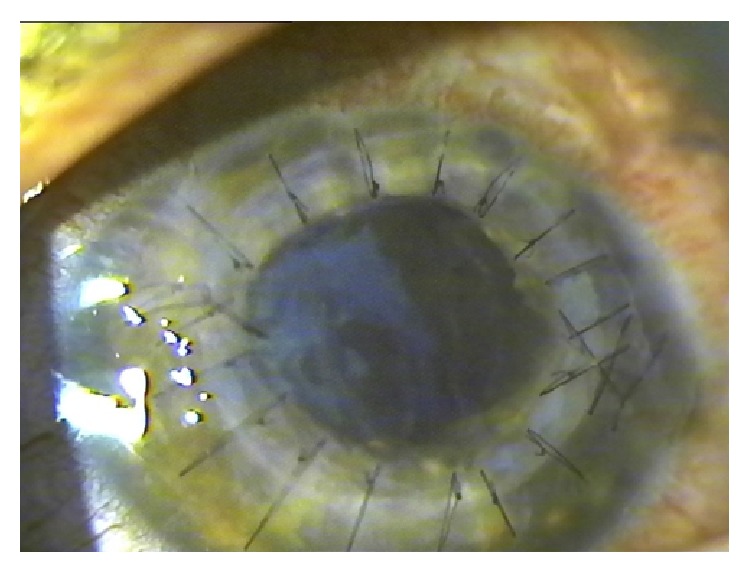
Graft failure after one year of the follow-up after combined surgery.

The corneal graft was transparent during the follow-up in 3 cases (25%) (Figure 8). Graft failure was observed in 9 eyes (75%) (Figure 9). Ahmed valve was implanted in two eyes due to uncontrolled glaucoma, cases 2 and 10. Overall, phthisis bulbi evolved in 2 cases (16%), cases 4 and 6; hypotony evolved in another 2 cases (16%), cases 3 and 11. Silicone oil was used as a tamponade in all cases; retina was reattached in 92% of cases. No eye was enucleated; no eye revealed signs of sympathetic ophthalmia. All eyes were silicon-oil sustained.

## 5. Discussion

Wide-field Landers TKP is a useful intraoperative tool to visualize the posterior segment even in case of large posttraumatic corneal wounds. By performing combined PPV/TKP/PKP procedure many maneuvers are possible during PPV: ILM peeling, vitreous shaving, and fluid-air and air-silicone-oil exchange, using perfluorocarbon liquids. Landers wide-field TKP is not leaking during indenting and is reusable.

Nevertheless, both functional and anatomical outcomes of our case series of 12 patients were not favorable probably because of severity of the initial trauma. However, the natural course of the disease would be much more worse resulting in phthisis bulbi in most of the cases.

The reason for these unsatisfactory results in our case series was mostly corneal graft failure (75%) or hypotony due to ciliary body dysfunction (16%) or glaucoma (16%).

All eyes in our study were silicone-oil sustained and it is known that silicone oil may stress the donor endothelium and may result in decompensation of the graft [9, 10]. Moreover, in eyes with long-term silicone oil tamponade, silicon oil may be present in the anterior chamber causing keratopathy by direct contact with corneal endothelium [5].

In other case series the percentage of eyes with clear corneas after longitudinal follow-up following combined PPV/TKP/PKP ranged from 15% [11] to 75% [12]. It has been already shown that the risk of corneal graft failure is increased if PKP is performed during the first 2 months after ocular trauma [11]. In our series the mean time from injury to PPV/TKP/PKP was 24 days. On the other side it is recommended to perform PPV after severe ocular trauma and retinal detachment in the first two weeks [13, 14]. Dong and colleagues [12] suggest performing this combined surgery within 1 month from injury. They reported results of 78 eyes operated with Landers keratoprosthesis after 24 months of observation. Unsatisfactory postoperative visual acuity in their study was due to graft failure, recurrent PVR, or secondary glaucoma.

In the literature, the final retinal reattachment varied from 43% [15] to 91% [16]. In our case series reattachment rate was quite high and amounted to 92%.

In the prediction of the good visual outcome after open-globe injury several factors have been identified: initial visual acuity [7], afferent pupillary defect, type of trauma, wound location, and retinal detachment [13]. Thus, it should be considered in patients with both anterior and posterior segment damage after severe eye trauma classified for combined surgical procedure.

Some authors [17] prefer to perform TKP-assisted PPV first and suture back the corneal button. They perform TKP later, after one year, as a second procedure in selected cases, when intraocular pressure is more than 8 mmHg. However, in a study of Chen et al. 13% of eyes were enucleated due to atrophia bulbi, 62% were silicone-oil sustained, 20% of eyes were anatomically restored and 4% evolved recurrent retinal detachment [17].

Chun et al. published a study comparing application of temporary keratoprosthesis and endoscopy [18]. Authors concluded that the major differences observed between the two techniques are that endoscopy allows earlier intervention and shorter surgical times than does TKP.

In conclusion, combined PPV/TKP/PKP provides the opportunity to salvage the eyes after severe ocular trauma. However, patients should be informed about the longitudinal results of this combined surgery and that visual recovery is rather poor. Careful selection of patients should be done in order to reduce the risk of complications.

## Figures and Tables

**Table 1:** Patients' characteristics: age, gender, type of injury, initial and final visual acuity, intraocular pressure, clarity of the corneal graft, attachment of the retina, complications, follow-up period, time from injury, and operation time.

Number	Gender	Age (years)	Type of injury, zone	Initial visual acuity, intraocular pressure	Final visual acuity, intraocular pressure	Clarity of the corneal graft, attachment of the retina	Complications	Follow-up period (months)	Days from injury	Surgical time (hours)
1	Male	59	Penetration, zone I	Counting fingers, 12 mmHg	Counting fingers to 50 cm, 14 mmHg	Opaque cornea, retina attached	Graft failure	14	24	3.5

2	Female	39	Penetration, zone I	Hand motion, 14 mmHg	Counting fingers to 30 cm, 20 mmHg	Clear cornea, retina attached	Secondary glaucoma, implantation of the Ahmed valve	18	30	3.0

3	Male	20	Penetration, zone II	Light perception, 8 mmHg	Counting fingers to 2 m, 9 mmHg	Opaque cornea, retina attached	Graft failure, hypotony, anterior synechiae	12	14	2.5

4	Male	71	Penetration, zone III	Hand motion, 16 mmHg	No light perception, 7 mmHg	Opaque cornea, retina attached	Phthisis bulbi	13	19	4.0

5	Male	43	Penetration, zone II	Hand motion, 17 mmHg	Hand motion, 13 mmHg	Opaque cornea, retinal detachment, PVR	Graft failure, anterior synechiae, retinal detachment	14	25	4.0

6	Male	48	Rupture, zone III	No light perception, 16 mmHg	No light perception, 14 mmHg	Opaque cornea, retina attached	Phthisis bulbi	13	23	3.5

7	Male	24	Intraocular foreign body, zone I	Hand motion hypo	Counting fingers to 70 cm, 14 mmHg	Cornea clear, retina attached	None	12	13	3.5

8	Male	64	Penetration, zone I	No light perception, 17 mmHg	No light perception, 15 mmHg	Opaque cornea, retina attached	Graft failure	29	45	4.0

9	Male	62	Intraocular foreign body, zone I	Hand motion, 12 mmHg	Counting fingers to 20 cm, 13 mmHg	Opaque cornea, retina attached	Graft failure	12	20	3.0

10	Female	21	Intraocular foreign body, zone II	No light perception, 13 mmHg	Counting fingers to 1 m, 25 mmHg	Opaque cornea, retina attached	Graft failure, secondary glaucoma, implantation of the Ahmed valve	24	14	2.5

11	Male	31	Intraocular foreign body, zone II	No light perception, 8 mmHg	No light perception, 7 mmHg	Clear cornea, retina attached	Hypotony	12	32	3.0

12	Male	22	Penetration, zone II	Light perception, 11 mmHg	Counting fingers, 12 mmHg	Opaque cornea, retina attached	Graft failure, anterior synechiae	16	24	3.0
